# Overgrowth of the lower limb after treatment of developmental dysplasia of the hip: incidence and risk factors in 101 children with a mean follow-up of 15 years

**DOI:** 10.1080/17453674.2019.1688485

**Published:** 2019-11-12

**Authors:** Chan Yoon, Chang Ho Shin, Dong Ook Kim, Moon Seok Park, Won Joon Yoo, Chin Youb Chung, In Ho Choi, Tae-Joon Cho

**Affiliations:** a Department of Orthopaedic Surgery, Seoul Bumin Hospital, Seoul;; b Division of Pediatric Orthopaedics, Seoul National University Children’s Hospital, Seoul;; c Department of Orthopaedic Surgery, Seoul National University Bundang Hospital, Seongnam, Gyeonggi, Republic of Korea

## Abstract

Background and purpose — There are few studies on overgrowth of the affected limb after treatment of developmental dysplasia of the hip (DDH). We investigated the incidence of overgrowth and its risk factors in DDH patients.

Patients and methods — 101 patients were included in this study. Overgrowth was defined by 2 criteria: when the height of the femoral head of the affected side was higher than that of the contralateral side by more than 10 mm, or by more than 15 mm. The potential risk factors of distinct overgrowth were retrospectively examined using multivariable analysis.

Results — When overgrowth was defined as femoral head height difference (FHHD) > 10 mm, its incidence was 44%, and only femoral osteotomy was identified as a significant risk factor with a relative risk (RR) of 1.6 (95% confidence interval [CI] 1.0–2.5). When overgrowth was defined as FHHD > 15 mm, its incidence was 23%, and femoral osteotomy was identified as the only significant risk factor with an RR of 2.3 (CI 1.2–4.5). Overgrowth developed more frequently in patients who underwent femoral osteotomy at the age of 2 to 4 years (87%) than in the others (46%) (p = 0.04).

Interpretation — Overgrowth of the affected limb is common in DDH patients. Patients who underwent femoral osteotomy, especially at the age of 2 to 4 years, may require careful follow-up because of the substantial risk for overgrowth.

Leg length discrepancy (LLD) sometimes occurs during the treatment of developmental dysplasia of the hip (DDH) (Kalamchi and MacEwen 1980, Porat et al. 1994, Zadeh et al. 2000, Inan et al. 2008). LLD may manifest as shortening of the affected limb from proximal femoral growth disturbance, or as overgrowth of the affected limb. Most previous studies focused on shortening due to proximal femoral growth disturbance (Kalamchi and MacEwen 1980, Porat et al. 1994, Inan et al. 2008). To our knowledge, only 1 study reported the incidence of overgrowth of the affected limb in patients with DDH (Zadeh et al. 2000). In that study, all hips that showed overgrowth of the affected limb by more than 15 mm had had a femoral osteotomy in conjunction with anterolateral open reduction.

Femoral osteotomy is performed to facilitate reduction, to correct excessive femoral anteversion, and to redirect the femoral head toward the acetabular center with intent to improve the stability of reduction, which is the primary stimulus for acetabular remodeling (Smith et al. 1963). However, femoral osteotomy also may risk overgrowth as a femoral shaft fracture (Staheli 1967, Zadeh et al. 2000).

Overgrowth and consequent LLD results in hip adduction and decrease of lateral center–edge angle on the long limb side in the weight-bearing position. This may lead to excessive load on the growth plate between the acetabular cartilage and the ilium and can consequently compromise normal acetabular development, resulting in the so-called “long-leg dysplasia” (Ponseti 1978, Zadeh et al. 2000).

We assessed the incidence and risk factors of LLD by overgrowth in patients who had been treated for DDH.

## Patients and methods

This was a retrospective cohort study. Medical records and serial radiographs of patients with DDH who were treated between April 1982 and December 2004 were reviewed. Inclusion criteria were dislocated-type DDH with unilateral involvement, which had not received any prior treatment before being referred to our hospital. Of 196 consecutive patients meeting these criteria, the following patients were excluded: patients who were not followed up until skeletal maturity (n = 68); patients associated with neuromuscular disease (n = 10); 1 patient with other congenital anomaly; and 4 patients who had medical conditions affecting leg length, such as septic arthritis of the hip. We also excluded 10 hips that presented after 5 years of age and 2 hips with type III osteonecrosis according to the criteria by Bucholz-Ogden (Roposch et al. 2012). Hips with type I or II osteonecrosis were included in the study. No hips had type IV osteonecrosis. Based on these criteria, 101 patients (101 hips) were enrolled in the study.

**Figure 1.**
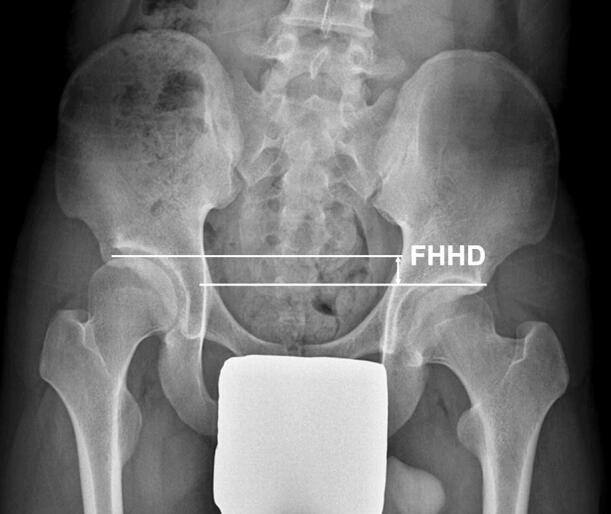
Measurement of femoral head height difference (FHHD) on standing anteroposterior radiograph of the hip.

LLD was determined on standing anteroposterior radiographs of the hip by measuring the femoral head height difference (FHHD) at skeletal maturity or at the time of intervention for overgrowth (Figure 1) (Friberg 1983). LLD was recorded as a positive value when the affected side was longer than the unaffected side. Distinct overgrowth was determined to be present with 2 criteria: FHHD > 10 mm or FHHD > 15 mm. It has been reported that LLD > 10 mm results in a significant mediolateral shift in the center of pressure toward the longer leg (Mahar et al. 1985, Gurney 2002).

Demographic data, initial severity of DDH, reduction method, osteotomy site, and deformity of proximal femur were considered candidate risk factors for overgrowth. Relative risk (RR) with 95% confidence interval (CI) was calculated and multivariable analysis was performed, respectively for the two definition of overgrowth. In order to evaluate initial severity, hips were graded according to the Tönnis classification (Tönnis et al. 1987), and the acetabular index (AI) was measured at the time of reduction. To evaluate deformity of proximal femur, osteonecrosis was classified according to Bucholz–Ogden criteria (Roposch et al. 2012), and the widest diameter of the femoral head was measured at skeletal maturity or just before the intervention for overgrowth. Coxa magna was recorded when the femoral head diameter of the affected side was larger by 10% than that of the unaffected side (Young et al. 2014). Skeletal maturity was determined based on the closure of the proximal femoral growth plate and triradiate cartilage.

Some patients had undergone repeated multiple osteotomies, and others had undergone both femoral and pelvic osteotomy, making the definition of “age at osteotomy” ambiguous. In turn, we did not include age at osteotomy in the multivariable analysis. In the subgroup of patients who underwent a single femoral or pelvic osteotomy, the association between age at osteotomy and development of distinct overgrowth was analyzed.

**Figure 2.**
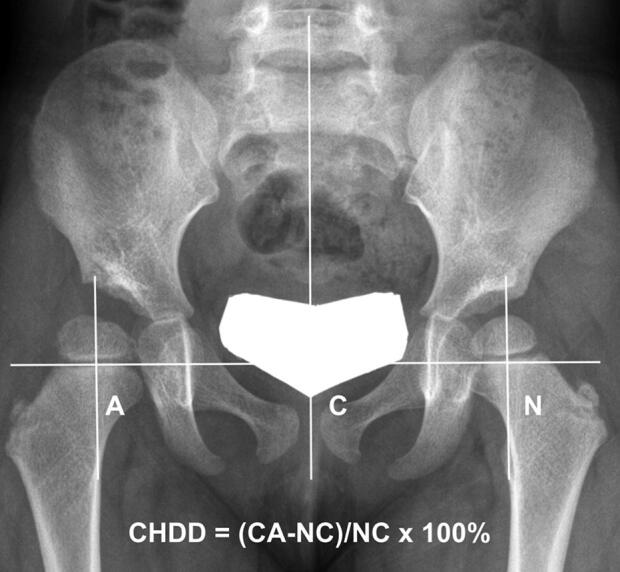
Measurement of center–head distance discrepancy (CHDD) on anteroposterior radiograph of the hip. The CHDD was defined as the difference in the center–head distance between the DDH side and the normal side, and expressed as a percentage of the normal side measurement.

Hip radiograph around 3 years of age (2 ∼ 4 years) was available in 42 of the 44 patients who did not undergo any osteotomy. The association between the AI and center–head distance discrepancy (CHDD) (Chen et al. 1994) around 3 years of age and development of overgrowth was analyzed in these patients (Figure 2). They were not measured in the osteotomy group because osteotomy was performed before 3 years of age in some patients and osteotomy could change those parameters.

All radiographs were reviewed by the 2 authors. To determine intra-observer reliability, measurements were made by the first author (CY) on 2 different days, 4 weeks apart. To determine the inter-observer reliability, the same measurements were made by another author (DOK) after a consensus-building session to define the radiographic measurements. Intra-observer and inter-observer reliability were evaluated by intraclass correlation coefficients (ICCs), which were calculated assuming absolute agreement and a single measurement with a 2-way random-effects model (see Appendix).

### Statistics

A sample size of 68 participants was required to detect a difference of 14% between groups in the incidence rate of LLD over 15 mm, using a 2-sided Z-test of the difference between proportions with a power of 80% at a level of significance of p < 0.05. This 14% difference represents the difference between a 20% LLD incidence rate in the DDH group and 6% rate in the normal population (Knutson 2005).

Continuous data were statistically analyzed using the independent Student t-test or Mann–Whitney U-test after the Kolmogorov–Smirnov normality test, and categorical data were analyzed using the chi-square test or Fisher’s exact test. Associations between risk factors and the development of distinct overgrowth were assessed using a log-binomial model to calculate adjusted RR and CI. Since the incidence of overgrowth was more than 10% in this study, we used the log-binomial model instead of logistic regression analysis to avoid overestimating the risk (McNutt et al. 2003). Univariable analysis was performed initially to assess baseline differences between patients with and without distinct overgrowth. Next, variable selection for multivariable analysis was based on a causal path diagram that was created using the directed acyclic graph (DAG) (Shrier and Platt 2008). Covariates in DAG were selected based on previous literature and hypothesized relationship (Kalamchi and MacEwen 1980, Tönnis et al. 1987, Zadeh et al. 2000, Hefti 2007). A receiver operating characteristic (ROC) curve was applied to determine cut-off values for the AI and CHDD at the age of 3 years, which distinguished between the cases with and without distinct overgrowth in the non-osteotomy group. P-values of < 0.05 were considered statistically significant.

### Ethics, funding, and potential conflicts of interest

This study was approved by the institutional ethics committee (H-1711-013-895) and was performed in accordance with the Declaration of Helsinki. No funding was received and there are no competing interests declared.

## Results

There were 91 female and 10 male patients. 59 hips were left-side hips. Preoperatively, 76 hips were Tönnis grade II, 16 hips grade III, and 9 hips grade IV. Various treatment modalities had been used (Table 1). Pre-reduction skin traction was used in 16 patients. The mean period of traction was 8 days (3–26). The mean age at the latest follow-up was 17 years (12–29), and the follow-up duration averaged 15 years (8–26). 5 hips had type I, and 29 hips had type II osteonecrosis.

**Table 1.** Treatment modalities applied to patients

Treatment modalities	Number of hips (N = 101)	Mean age (SD) at treatment
Closed reduction (CR)	34	14 (6) months
CR with femoral osteotomy	1	36 months
Medial open reduction (OR)	10	13 (5) months
Anterolateral OR **^a^**	33	17 (9) months
with femoral osteotomy	5	19 (3) months
with pelvic osteotomy **^b^**	14	27 (15) months
with femoral and pelvic osteotomies	4	28 (10) months
Osteotomy for residual dysplasia		
Femoral osteotomy **^c^**	10	3.3 (2.0) years
Pelvic osteotomy **^d^**	10	4.8 (2.8) years
Femoral and pelvic osteotomies **^e^**	13	
femoral		4.2 (2.6) years
pelvic		4.5 (2.7) years

**^a^**2 hips had been redislocated after CR.

**^b^**1 hip had been redislocated after CR and 1 hip after anterolateral OR.

**^c^**There were patients who had repeated femoral osteotomies (twice, n = 2; 3 times, n = 1) before skeletal maturity.

**^d^**1 patient had had pelvic osteotomy twice before skeletal maturity.

**^e^**There were patients who had repeated femoral osteotomies (twice, n = 1; 3 times, n = 1) or repeated pelvic osteotomies (twice, n = 1) before skeletal maturity.

CR = closed reduction under general anesthesia; OR = open reduction.

FHHD was more than 10 mm in 44 patients (95% CI 35–53) and more than 15 mm in 23 patients (CI 16–29) (Table 2). 24 patients underwent intervention for LLD. 16 patients had epiphysiodesis in the distal femur at a mean age of 11.6 years (10.7–12.6), and 8 patients had femoral shortening combined with varization osteotomy at a mean age of 7.9 years (3.4–12.5). Their mean FHHD was 13 mm (10–19) at surgical intervention and 1 mm (–15 to 13) at skeletal maturity.

**Table 2.** Femoral head height difference (FHHD) in the patients with distinct overgrowth. Values are number of hips

FHHD, mm	Intervention for overgrowth **^a^** (n = 24)	No intervention for overgrowth **^b^** (n = 20)	Total
> 20	3	2	5
> 15	14	9	23
> 10	24	20	44

**^a^**FHHD was measured at intervention.

**^b^**FHHD was measured at skeletal maturity.

In the univariable analysis, anterolateral OR and femoral osteotomy were significant risk factors in both definitions of distinct overgrowth (Table 3, see Supplementary data). On the basis of the DAG and our univariable analysis, the following variables were included in the relevant multivariable analysis: age at reduction, initial severity, reduction method, and femoral osteotomy.

**Table 4.** Multivariable analysis of risk factors for development of overgrowth of the affected limb in overall patients

	FHHD > 10 mm		FHHD > 15 mm	
Risk factors	RR (95% CI)	p-value	RR (95% CI)	p-value
Age at reduction	1.0 (1.0–1.0)	0.8	1.0 (1.0–1.1)	0.6
Initial severity				
Tőnnis grade ≥ III	1.0 (0.6–1.5)	0.8	0.8 (0.4–1.7)	0.6
AI at reduction	1.0 (1.0–1.0)	0.6	0.9 (0.9–1.0)	0.1
Reduction method				
Anterolateral OR	1.6 (1.0–2.8)	0.08	2.4 (1.0–5.9)	0.06
Femoral osteotomy	1.6 (1.0–2.5) **^a^**	0.03	2.3 (1.2–4.5) ^a^	0.02

**^a^**Statistically significant.

FHHD = femoral head height difference; RR = relative risk;

CI = confidence interval; AI = acetabular index; OR = open reduction.

When distinct overgrowth was defined as FHHD > 10 mm or FHHD > 15 mm, only femoral osteotomy was found to be a significant risk factor with a RR of 1.6 (CI, 1.0–2.5) or a RR of 2.3 (CI, 1.2–4.5), respectively, according to multivariable analysis (Table 4).

Demographic and clinical characteristics of femoral osteotomy and non-femoral osteotomy groups were comparable except for the proportion of hips with coxa magna (Table 5, see Supplementary data). Of 33 patients in the femoral osteotomy group, 31 patients underwent femoral varization derotational osteotomy, and 2 patients underwent femoral derotational osteotomy. Neck shaft angle of the affected side was 153° (SD 7°) preoperatively, 135° (10°) immediately after femoral osteotomy, and 135° (6°) at skeletal maturity or at the time of intervention for overgrowth. The neck shaft angle of the contralateral side was 151° (10°), 152° (8°), and 135° (6°), respectively. Distinct overgrowth developed more frequently in the femoral osteotomy group than in the non-femoral osteotomy group (Table 5, see Supplementary data). In 28 patients who underwent a single femoral osteotomy, distinct overgrowth developed much more frequently in patients who underwent femoral osteotomy at the age of 2 to 4 years (13/15) than those who underwent femoral osteotomy before the age of 2 years (2/5) or after the age of 4 years (4/8) (p = 0.04).

In the non-osteotomy group, the CHDD at the age of 3 years was significantly larger in the overgrowth group than in the no-overgrowth group when distinct overgrowth was defined as FHHD > 10 mm (p = 0.005), while it was not when it was defined as FHHD > 15 mm (Table 6, see Supplementary data). The AI at the age of 3 years was not significantly different between the overgrowth and no-overgrowth groups in both definitions of distinct overgrowth. An ROC curve showed the optimal cutoff value for distinct overgrowth (FHHD > 10 mm) to be a CHDD of 7%, with 77% sensitivity and 76% specificity (area under the curve = 0.8, CI 0.6–0.9; p = 0.009). The incidence of distinct overgrowth (FHHD > 10 mm) was higher in patients with a CHDD of > 7% (10/17) than patients with a CHDD of ≤ 7% (3/25) in the non-osteotomy group (p < 0.002).

## Discussion

Little has been reported on the incidence and risk factors of LLD by overgrowth in patients with DDH. In the current study, more than 40% of patients treated by closed reduction (CR) or open reduction (OR) had LLD exceeding 10 mm. LLD of 10 mm may not have a considerable influence on normal hips (Song et al. 1997). However, in patients with DDH, a small amount of overgrowth might compromise development of the acetabulum, which is already dysplastic, by increased mechanical compression of the growth plate of the acetabular cartilage complex and Hueter–Volkmann law (Ponseti 1978, Stokes 2002). It can break the balance between the growth of the acetabular and triradiate cartilages, which is important for normal acetabular development to occur as the pelvis enlarges (Ponseti 1978).

In our study cohort, overgrowth > 10 mm was observed in 44% of patients, and > 15 mm in 23%. This incidence is much higher than that of a healthy cohort of 600 military recruits, 4% of whom had an LLD of more than 15 mm (Hellsing 1988). Our results are similar to a previous study reporting an incidence of 17% of overgrowth more than 15 mm, and recurrence of hip dysplasia in 5 of 12 hips with an increase in leg length (Zadeh et al. 2000).

**Figure 3.**
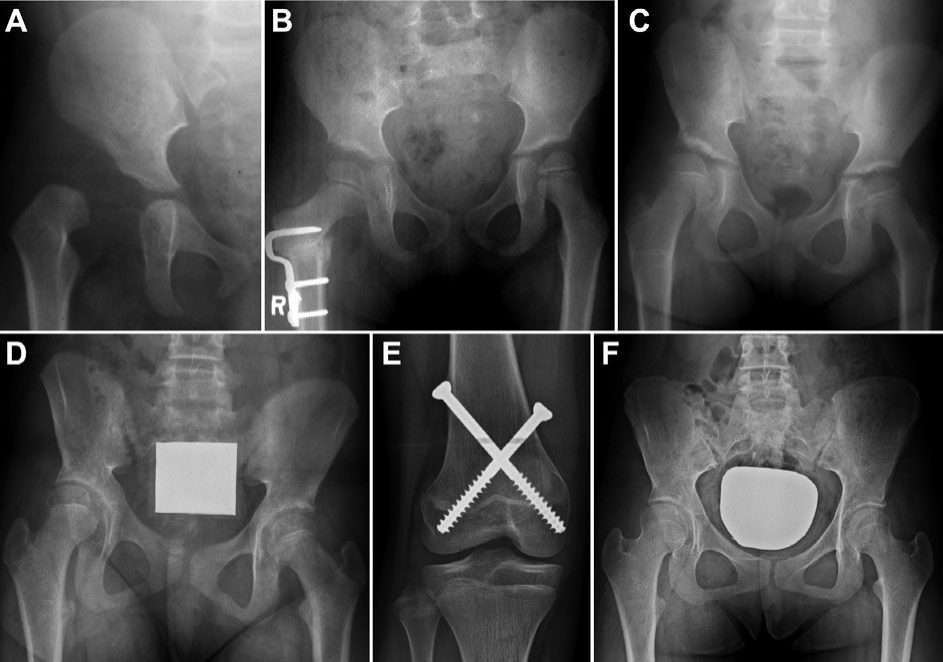
An example of overgrowth of the affected limb after treatment of DDH. A girl underwent anterolateral open reduction at age 1.5 years (A) and femoral osteotomy at age 5 years. At 2 months post-osteotomy FHHD was not distinct (B). However, FHHD became +14 mm at age 8.5 years (C) and +19 mm at age 11.5 years (D) resulting in pelvic tilt. She underwent percutaneous epiphysiodesis using transphyseal screws (E) and eventually had a level pelvis at age 15 years (F).

We found femoral osteotomy to be an independent risk factor for overgrowth after adjusting for other risk factors. Similar to our results, Zadeh et al. (2000) reported that all the hips that showed overgrowth after OR for DDH had undergone femoral osteotomy. Geometrically, proximal femoral varus osteotomy shortens the effective length of the femur (Suda et al. 1995). However, we found that the affected leg showed overgrowth after femoral varus osteotomy and eventually became longer than the unaffected leg at skeletal maturity or at the time of intervention for overgrowth (Figure 3). This justifies the intentional shortening of the effective femur length using the medial closing-wedge technique of varus osteotomy, and further shortening by trapezoidal wedge resection may be considered. This overgrowth phenomenon may share the same pathogenic mechanism with overgrowth after femoral shaft fracture (Staheli 1967, Shapiro 1981, Corry and Nicol 1995). Many studies reported that it occurs mainly in children over 2 years of age (Staheli 1967, Corry and Nicol 1995). In accordance with results of these studies, overgrowth in our DDH cohort occurred more often when femoral osteotomy was performed at the age of 2 to 4 years. In contrast, Suda et al. (1995) reported no difference in femoral length between the affected and unaffected sides at skeletal maturity after femoral varus osteotomy in DDH patients. However, they evaluated LLD in only 45% of the 42 subjects due to the unavailability of radiographs, and their mean age at femoral osteotomy was 4.7 years, which was older than the most vulnerable age for overgrowth in our study. This finding suggests that the risk of overgrowth should be considered when performing femoral osteotomy, especially at the age of 2 to 4 years.

In the non-osteotomy group, large CHDD around 3 years of age was associated with FHHD > 10 mm. Although it failed to show a statistically significant association with FHHD > 15 mm may be due to type II error, hips with overgrowth had larger mean CHDD than hips with no overgrowth around 3 years of age. It is difficult to speculate its pathogenic mechanism. A study on adult hip dysplasia showed that two-thirds of patients who did not undergo any surgery during childhood had an affected leg longer than the unaffected leg by more than 5 mm (Metcalfe et al. 2005). Altered mechanical loading on the proximal femur by lateral subluxation, which appeared as large CHDD, might affect leg length through the Hueter–Volkmann law (Stokes 2002). We could not exclude the possibility that LLD persisted in early childhood before measuring CHDD because standing hip radiographs could not be taken in early childhood and whole-leg radiograph was not routinely taken during follow-up.

Before the commencement of this study, we had an impression that anterolateral OR is an independent risk factor for overgrowth. In a previous study, which did not adjust confounding variables, all hips that showed overgrowth underwent femoral osteotomy in conjunction with anterolateral OR (Zadeh et al. 2000). In our study, anterolateral OR was a statistically significant risk factor in univariable analysis but showed borderline significance in multivariable analysis after adjusting for other variables, such as performance of osteotomy.

In our study, occurrence of type II osteonecrosis was not associated with overgrowth. It could be partly because the deformity in type II osteonecrosis is caput valgum rather than coxa valga and the center of rotation is close to the top of the femoral head (Shin et al. 2017). Moreover, severe type II osteonecrosis shortens the femoral neck, which may compensate for the lengthening effect of the proximal femoral valgus.

Our study has several limitations. First, LLD measured by iliac crest height difference better reflects pelvic tilt and its influence on the spine compared with LLD measured by FHHD. However, we had no choice but to measure FHHD because this study was a retrospective study and the iliac crest was not covered in many radiographs; this may be the reason why pelvic osteotomy was not a significant risk factor for overgrowth in this study. In addition, whole-leg radiographs were not available in many cases, which was also due to the retrospective design of this study. Therefore, although we hypothesize that LLD was attributable to femoral overgrowth rather than tibial overgrowth in most cases, we could not prove it. Second, there may be a selection bias in estimating the incidence of overgrowth in our DDH cohort. Those who had risk factors for overgrowth, such as femoral osteotomy and large CHDD, tended to be more compliant in terms of clinical visits compared with those who showed uneventful hip joint development. By the same token, those treated by a Pavlik harness were not followed up until skeletal maturity and were not included in this study.

Despite these limitations, we conclude that overgrowth of the affected limb is a commonly encountered problem after DDH treatment. DDH patients who had undergone femoral osteotomy, especially between the ages of 2 to 4 years, and those who have a large CHDD around 3 years of age, require careful follow-up for LLD development because it may jeopardize normal acetabular development. Further studies are warranted to prove the association between overgrowth of the affected leg and recurrence of hip dysplasia.

## Supplementary data

Appendix and Tables 3, 5, and 6 are available as supplementary data in the online version of this article, http://dx.doi.org/10.1080/17453674.2019.1688485

## Supplementary Material

Supplemental Material
