# Supplementary material for: Overgrowth of the lower limb after treatment of developmental dysplasia of the hip: incidence and risk factors in 101 children with a mean follow-up of 15 years
Source: Acta Orthop. 2019 Nov 12;91(2):197–202. doi: 10.1080/17453674.2019.1688485 (PMC7144334; doi:10.1080/17453674.2019.1688485)
Supplement: Supplemental Material [file IORT_A_1688485_SM0874.pdf]

Supplementary data

Appendix. Intraclass correlation coefficients calculated to evaluate intraobserver and interobserver reliability

Parameters	Intraobserver (95% CI)	Interobserver (95% CI)
FHHD	0.998 (0.997–0.999)	0.996 (0.994–0.997)
AI	0.895 (0.783–0.950)	0.821 (0.731–0.881)
CHDD	0.961 (0.920–0.981)	0.915 (0.874–0.943)
Femoral head diameter	0.919 (0.830–0.961)	0.904 (0.799–0.955)

FHHD = femoral head height difference; AI = acetabular index; CHDD = center–head distance discrepancy; CI = confidence interval.

Table 3. Univariable analysis of risk factors for development of overgrowth of the affected limb in overall patients. Values are number of hips unless otherwise specified

	FHHD > 10 mm				FHHD > 15 mm			
Risk factors	Overgrowth (n = 44)	No overgrowth (n = 57)	RR (95% CI)	p-value	Overgrowth (n = 23)	No overgrowth (n = 78)	RR (95% CI)	p-value
Age at reduction, months, mean (SD)	18 (9)	17 (11)	1.0 (1.0–1.0)	0.8	19 (10)	17 (10)	1.0 (1.0–1.0)	0.7
Male sex	7	3	2.0 (0.8–5.2)	0.1	5	5	2.5 (1.2–5.3) [a]	0.02
Initial severity								
Tönnis grade ≥ III	12	13	1.1 (0.7–1.7)	0.7	6	19	1.1 (0.5–2.5)	0.8
AI at reduction (°), mean (SD)	33 (6)	33 (6)	1.0 (1.0–1.0)	0.7	32 (6)	34 (6)	1.0 (0.9–1.0)	0.2
Reduction method								
Anterolateral OR	30	26	1.5 (1.1–2.1) [a]	0.03	18	38	2.9 (1.2–7.2) [a]	0.02
Osteotomy site								
Femoral osteotomy	21	12	1.8 (1.1–2.9) [a]	0.003	13	20	2.7 (1.3–5.5) [a]	0.007
Pelvic osteotomy	19	22	1.1 (0.8–1.6)	0.6	12	29	1.6 (0.8–3.3)	0.2
Deformity of proximal femur								
Type II osteonecrosis	13	16	1.0 (0.7–1.5)	0.9	6	23	0.9 (0.4–2.0)	0.8
Coxa magna	16	12	1.4 (0.9–2.3)	0.07	12	16	2.8 (1.4–5.7) [a]	0.003

[a] Statistically significant.
FHHD = femoral head height difference; RR = relative risk; CI = confidence interval; AI = acetabular index; OR = open reduction.

Table 5. Demographic and clinical characteristics of femoral osteotomy and non-femoral osteotomy groups. Values are number of hips unless otherwise specified

Characteristics	Femoral osteotomy group (n = 33)	Non-femoral osteotomy group (n = 68)	p-value
FHHD > 10 mm	21	23	0.01 [b]
FHHD > 15 mm	13	10	0.01 [b]
Age at reduction, month [a]	19 (8)	17 (11)	0.3 [c]
Female sex	29	62	0.7 [c]
Tönnis grade ≥ III	9	14	0.5 [b]
AI at reduction (°) [a]	34 (6)	33 (6)	0.7 [c]
Anterolateral OR	22	34	0.1 [b]
Pelvic osteotomy	17	24	0.1 [b]
Type II osteonecrosis	10	19	0.8 [b]
Coxa magna	14	14	0.03 [b]

[a] The values are given as the mean (SD).
[b] Fisher's exact test.
[c] Student's t-test.
FHHD = femoral head height difference; AI = acetabular index; OR = open reduction

Table 6. Comparison of the acetabular index (AI) and center–head distance discrepancy (CHDD) at the age of 3 years between the overgrowth and no-overgrowth groups in 42 hips that had not undergone femoral or pelvic osteotomies. Values are mean (SD)

Radiographic parameters at age 3 years	Overgrowth (n = 13)	No overgrowth (n = 29)	p-value [a]
FHHD > 10 mm			
AI (°)	27 (5)	25 (6)	0.3
CHDD (%)	10 (6)	6 (4)	0.005
FHHD > 15 mm			
AI (°)	25 (5)	26 (6)	0.8
CHDD (%)	10 (5)	7 (5)	0.2

[a] Mann-Whitney U-test.
FHHD = femoral head height difference.
